# A Concise Synthesis of Sacidumlignan B

**DOI:** 10.3390/molecules27185775

**Published:** 2022-09-07

**Authors:** Zhiyuan Zhuang, Zhenbiao Luo, Sichen Yao, Yawen Wang, Yu Peng

**Affiliations:** 1 State Key Laboratory of Applied Organic Chemistry, College of Chemistry and Chemical Engineering, Lanzhou University, Lanzhou 730000, China; 2 Sichuan Engineering Research Center for Biomimetic Synthesis of Natural Drugs, School of Life Science and Engineering, Southwest Jiaotong University, Chengdu 610031, China

**Keywords:** cyclolignan, total synthesis, cyclization, synthetic efficiency, Barbier reaction

## Abstract

A short synthesis of racemic Sacidumlignan B was achieved for the first time. The key steps included a formal reductive coupling between the diaryl ketone and the crotyl bromide, and the subsequent Friedel–Crafts cyclization, which led to an efficient construction of dihydronaphthalene skeleton in this 2,7′-cyclolignan natural product.

## 1. Introduction

**Figure 1:**
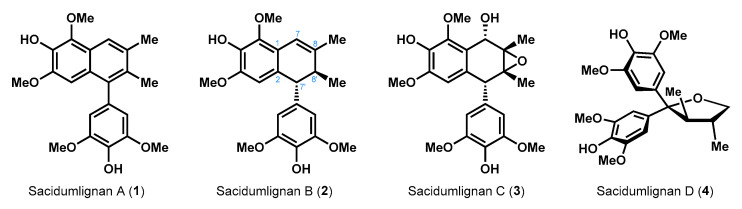
The structures of Sacidumlignans A–D.

Yue and co-workers isolated Sacidumlignans A–D (**1**–**4**, Figure 1) from the EtOH extract of *Sarcostemma acidum* (Roxb.) in Hainan Island of China [1]. Among these natural products, Sacidumlignans A–C (**1**–**3**) belong to typical 2,7′-cyclolignans, which have already attracted broad attention from the synthetic community [2,3]. Previously, Ramana et al. realized the asymmetric synthesis of Sacidumlignan B (**2**), a 7′,8′-dihydronaphthalene member, in 14 steps and 10% overall yield from *o*-vanillin [4]. Later, we achieved a unified synthesis of Sacidumlignans A (**1**) and D (**4**) by using Ueno–Stork radical cyclization reaction [5]. Recently, we also developed a base-promoted addition of *N*,*N*-dimethylacetamide to 1,1-diarylethylenes, and successfully applied this method to the total synthesis of (−)-Sacidumlignan B (**2**) in 3% overall yield [6]. However, this route was 16 steps, and therefore more efficient strategy for total synthesis of Sacidumlignan B is still in high demand. Herein, the first racemic synthesis of this molecule (**2**), featuring seven steps (46% overall yield) from a known compound, was demonstrated by our group (38% overall yield, nine steps from commercially available syringol/syringaldehyde).

## 2. Results and Discussion

### 2.1. Retrosynthetic Analysis of Sacidumlignan B

**Scheme 1:**
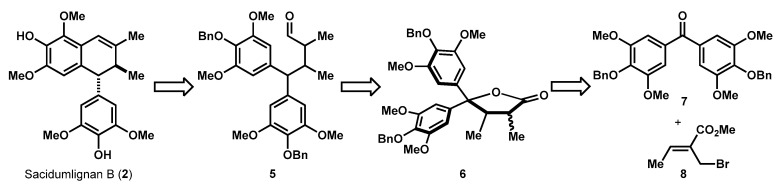
Retrosynthetic Analysis of Sacidumlignan B.

As shown in Scheme 1, this step-economic synthesis of Sacidumlignan B (**2**) would end from aldehyde **5** by the Friedel–Crafts cyclization and subsequent dehydration under acidic condition. This aldehyde would then be generated from lactone **6** through a redox manipulation. Eventually, lactone **6** could be prepared from known diaryl ketone **7** [5] by a formal reductive coupling with crotyl ester **8**.

### 2.2. Facile Construction of Sacidumlignan B Skeleton

**Scheme 2:**
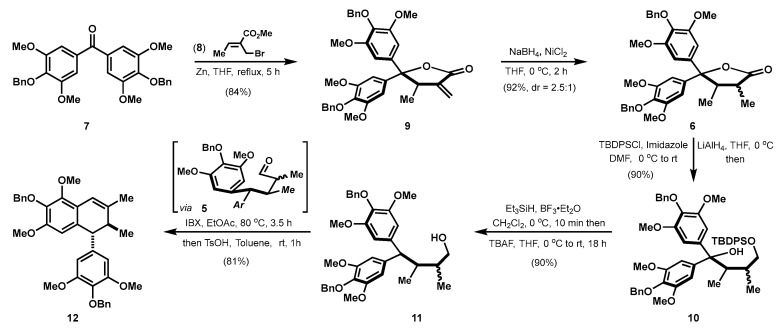
Construction of Dihydronaphthalene skeleton of Sacidumlignan B.

In a forward manner (Scheme 2), this synthesis began with a zinc-mediated Barbier reaction of diaryl ketone **7** and crotyl bromide **8** [7]. This reaction still proceeded well in gram-scale, and the generated intermediate spontaneously cyclized to form α-*exo*-methylene-γ-lactone **9** in one-step and 84% yield. With all the carbon atoms of Sacidumlignan B in hand, the next redox reactions were carried out. First of all, the double bond in **9** was reduced. The NaBH_4_ and NiCl_2_ combination in icy THF proved to be an optimal condition [8], and α,β-dimethyl-γ-lactone **6** as the inconsequential diastereomers (dr = 2.5:1) was thus obtained in 92% yield. The subjection of **6** to the reduction by LiAlH_4_ followed by a selective *tert*-butyldiphenyl silyl (TBDPS) protection of the resultant diol, afforded monoalcohol **10** in 90% yield. The remaining tertiary alcohol was then reduced by Et_3_SiH under the assistance of BF_3_∙Et_2_O [9], and the subsequent deprotection of TBDPS group in the resulting silyl ether delivered the primary alcohol **11** in 90% yield. Oxidation of this alcohol by IBX followed by acid-promoted dehydrative Friedel-Crafts cyclization of the generated aldehyde **5** with a pseudo-half-chair conformation where aryl and the adjacent methyl are oriented at respective equatorial position, eventually established the *trans*-7′,8′-dihydronaphthalene skeleton with a diarylmethine stereocenter [10] and therefore gave benzyl-protected Sacidumlignan B (**12**) in 81% overall yield.

### 2.3. Investigation to Debenzylation

The end game of total synthesis for Sacidumlignan B (**2**) seems to the only removal of two benzyl groups in **12**, which proved to be problematic at the initial investigations (Table 1). Pd/C-catalyzed hydrogenation at room temperature in EtOH led to not only the deprotection of benzyl groups, but also over-reduction in the double bond in **12**, therefore providing dihydroSacidumlignan B (**13**, dr = 8:1) in almost quantitative yield (entry 1). Utilization of Lewis acids was next evaluated. The condition with either AlCl3 [11] or FeCl3 [12] gave no **2** and **13** (entries 2 and 3). To our delight, subjection of **12** into five equivalents of TiCl4 in DCM at 0 °C [13] resulted in the production of the desired Sacidumlignan B (**2**), albeit in 15% yield (entry 4). Allowing the reaction temperature to be lowered to −45 °C can provide a significant improvement of the yield (47%, entry 5), and further lowering temperature led to a slight decrease in the yield for **2** instead (entry 6). Employment of BCl_3_ could also afford the desired **2** in 49% yield. Anyway, the total synthesis of Sacidumlignan B (**2**) was achieved eventually in 23% overall yield from diaryl ketone **7**.

### 2.4. Efficient Synthesis of Sacidumlignan B

**Scheme 3:**
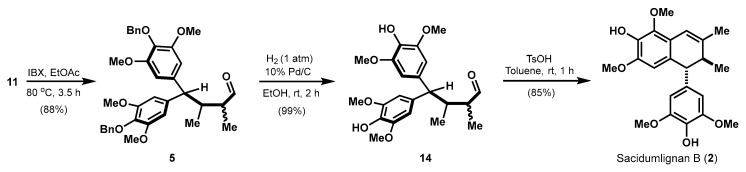
Completion of synthesis for Sacidumlignan B.

In order to pursue higher efficiency for the synthesis of Sacidumlignan B (**2**), a modification of the reaction sequence was carried out (Scheme 3). To this end, aldehyde **5** already obtained in Scheme 1 was hydrogenated first to remove benzyl groups, generating phenol **14** in almost quantitative yield. The similar acid-promoted dehydrative Friedel–Crafts cyclization to that of **5** to **12** proceeded smoothly, and 85% yield of Sacidumlignan B (**2**) could be produced. This adjusted route can provide a two-fold increase (46% vs. 23%) in overall yield for Sacidumlignan B (**2**), further demonstrating significant superiority to the previous syntheses [4,6].

## 3. Materials and Methods

### 3.1. General Procedure

For product purification by flash column chromatography, silica gel (200~300 mesh) and petroleum ether (bp. 60~90 °C) were used. All solvents were purified and dried by standard techniques and distilled prior to use. All of the experiments were conducted under an argon or nitrogen atmosphere in oven-dried or flame-dried glassware with magnetic stirring, unless otherwise specified. Organic extracts were dried over Na_2_SO_4_, unless otherwise noted. ^1^H and ^13^C NMR spectra were taken on a *Bruker* AM-400 (Bruker, Romanshorn, Switzerland) with TMS as an internal standard and CDCl_3_ as solvent unless otherwise noted.

### 3.2. Synthesis of Compound **9**

Compound **8** was prepared according to a known procedure [14].

To a mixture of acetaldehyde (5.7 mL, 100 mmol) and methyl acrylate (13.5 mL, 150 mmol, 1.5 equiv) in a 50-mL, two-necked, round-bottom flask was added DABCO (1.68 g, 15 mmol). The resulting mixture was stirred at room temperature for 7 days. After completion, the reaction mixture was treated with 10% HCl (aq.) and Et_2_O (100 mL) was then added. The organic phase was separated, washed by water (2 × 8 mL), brine (8 mL), dried over anhydrous Na_2_SO_4_ and concentrated in vacuo to afford the desired alcohol (9.4 g) as a pale-yellow oil. This crude allyl alcohol could be used directly without further purification.

To a solution of the above allyl alcohol (9.4 g, 72.3 mmol) in anhydrous Et_2_O (50 mL) was added phosphorus tribromide (7.7 mL, 86.8 mmol, 1.2 equiv) dropwise at 0 °C. The resulting mixture was stirred at 0 °C for 2 h. After the reaction was completed, the mixture was poured into ice water, extracted with Et_2_O (150 mL). The combined organic layers were washed with saturated NaHCO_3_ solution (20 mL), water (2 × 10 mL), brine (10 mL), dried over anhydrous Na_2_SO_4_ and concentrated in vacuo. The residue was purified by flash column chromatography (petroleum ether/EtOAc = 30:1 → 10:1) on silica gel to afford bromide **8** (11.6 g, 86% yield) as a colorless oil.

A dry 100 mL two-necked, round-bottom flask equipped with a reflux condenser were charged with activated zinc powder (780 mg, 12 mmol, 1.2 equiv) and THF (20 mL) under argon. The allyl bromide **8** (4.8 g, 25 mmol, 2.5 equiv) and diaryl ketone **7** (5.15 g, 10 mmol) were then added to the above smoothly stirred system. The generated mixture was stirred for 5 min at room temperature then heated to 70 °C for 5 h until TLC indicated the reaction was completed. Then the reaction was cooled to room temperature and quenched by saturated NH_4_Cl solution (2 mL). The mixture was diluted with EtOAc (150 mL), and the organic layers were washed with water (2 × 10 mL), brine (10 mL), dried over anhydrous Na_2_SO_4_, and concentrated under reduced pressure. The residue was purified by flash column chromatography (petroleum ether/EtOAc = 8:1 → 4:1) on silica gel to afford γ-lactone **9** (4.99 g, 84% yield) as a colorless oil. *R_f_* = 0.41 (petroleum ether/EtOAc = 4:1); ^1^H NMR (400 MHz, CDCl_3_): *δ* = 7.47 (d, *J* = 7.2 Hz, 2H), 7.43 (d, *J* = 7.2 Hz, 2H), 7.33–7.24 (m, 6H), 6.70 (s, 2H), 6.28 (d, *J* = 2.4 Hz, 1H), 6.27 (s, 2H), 5.55 (d, *J* = 2.4 Hz, 1H), 5.04 (s, 2H), 5.00 (s, 2H), 3.79 (s, 6H), 3.78–3.74 (m, 1H), 3.68 (s, 6H), 1.02 (d, *J* = 6.8 Hz, 3H) ppm; ^13^C NMR (100 MHz, CDCl_3_): *δ* = 169.2, 153.0 (2C), 152.8 (2C), 140.6, 137.9, 137.3 (2C), 136.6, 136.0, 135.8, 128.1 (4C), 127.8 (2C), 127.7 (2C), 127.6, 127.5, 121.2, 104.2 (2C), 103.6 (2C), 90.2, 74.6, 74.5, 56.1 (2C), 55.8 (2C), 43.6, 16.7 ppm. Copies of ^1^H and ^13^C NMR could be found in Supplementary Materials.

### 3.3. Synthesis of Compound **6**

NiCl_2_ (162 mg, 1.25 mmol, 0.25 equiv) was added to a solution of **9** (2.98 g, 5 mmol) in THF (20 mL) at 0 °C, and the resulting mixture was then stirred vigorously followed by the addition of NaBH_4_ (284 mg, 7.5 mmol, 1.5 equiv) portionwise. The reaction mixture was stirred at 0 °C for 2 h, and quenched with 10% HCl (2 mL). The mixture was diluted with EtOAc (60 mL), and the organic layers were washed with water (3 × 5 mL), brine (5 mL), dried over anhydrous Na_2_SO_4_ and concentrated under reduced pressure. The residue was purified by flash column chromatography (petroleum ether/EtOAc = 4:1) on silica gel to afford α-methylene-γ-lactone **6** (2.75 g, 92% yield) as a colorless oil. (*major isomer*) *R_f_* = 0.47 (petroleum ether/EtOAc = 2:1); ^1^H NMR (400 MHz, CDCl_3_): *δ* = 7.48–7.43 (m, 4H), 7.36–7.27 (m, 6H), 6.65 (s, 2H), 6.24 (s, 2H), 5.04 (s, 2H), 5.01 (s, 2H), 3.81 (s, 6H), 3.70 (s, 6H), 2.89–2.81 (m, 1H), 2.44–2.36 (m, 1H), 1.28 (d, *J* = 7.2 Hz, 3H), 1.05 (d, *J* = 6.4 Hz, 3H) ppm; ^13^C NMR (100 MHz, CDCl_3_): *δ* = 178.4, 153.3 (2C), 153.0 (2C), 138.4, 137.6 (2C), 137.0, 136.3, 135.6, 128.41 (2C), 128.38 (2C), 128.1 (2C), 128.0 (2C), 127.9, 127.8, 104.7 (2C), 104.1 (2C), 90.6, 74.9, 74.8, 56.4 (2C), 56.2 (2C), 46.3, 41.1, 16.2, 13.2 ppm.

### 3.4. Synthesis of Compound **10**

To a stirred solution of γ-lactone **6** (1.20 g, 2 mmol) in anhydrous THF (20 mL) at 0 °C under argon was added LiAlH_4_ (91 mg, 2.4 mmol, 1.2 equiv) portionwise. The reaction mixture was stirred for 3 h at this temperature and quenched carefully by ice water (2 mL) followed by the addition of 10% HCl (5 mL) dropwise. After stirring for 2 min, the resulting mixture was diluted with EtOAc (80 mL), and the organic layers were washed with water (5 × 8 mL) and brine (8 mL), dried over Na_2_SO_4_, filtered and concentrated under reduced pressure. The generated diol (1.18 g, 1.9 mmol) was dissolved in anhydrous DMF (10 mL) and cooled to 0 °C. Imidazole (264 mg, 3.8 mmol, 2 equiv) was then added and stirring was continued for 5 min followed by the addition of a solution of TBDPSCl (640 mg, 2.8 mmol, 1.2 equiv) in DMF (3 mL). The resulting mixture was warmed to 25 °C and stirred for 10 h. The reaction mixture was then diluted with EtOAc (150 mL) and poured into a separatory funnel. The organic layers were washed with water (5 × 15 mL) and brine (15 mL), dried over Na_2_SO_4_, filtered and concentrated under reduced pressure. The residue was purified by flash column chromatography (petroleum ether/EtOAc = 2:1) on silica gel to afford the tertiary alcohol **10** (1.55 g, 90% yield) as a colorless oil. (*major isomer*) *R_f_* = 0.34 (petroleum ether/EtOAc = 2:1); ^1^H NMR (400 MHz, CDCl_3_): *δ* = 7.69–7.67 (m, 2H), 7.53–7.45 (m, 7H), 7.44–7.38 (m, 4H), 7.35–7.27 (m, 7H), 6.90 (s, 2H), 6.88 (s, 2H), 5.76 (s, 1H), 5.03 (s, 2H), 4.97 (s, 2H), 3.82 (s, 6H), 3.75 (s, 6H), 3.51 (t, *J* = 10.8 Hz, 1H), 3.23 (dd, *J* = 10.8, 3.2 Hz, 1H), 2.60 (q, *J* = 6.8 Hz, 1H), 2.21–2.12 (m, 1H), 1.11 (s, 9H), 0.87 (d, *J* = 6.8 Hz, 3H), 0.75 (d, *J* = 7.2 Hz, 3H) ppm; ^13^C NMR (100 MHz, CDCl_3_): *δ* = 153.1 (2C), 152.8 (2C), 144.7, 143.0, 138.1, 138.0, 135.6 (2C), 135.5 (2C), 135.3, 135.2, 132.14, 132.09, 130.04, 129.96, 128.37 (2C), 128.35 (2C), 128.05 (2C), 128.03 (2C), 127.9 (2C), 127.8 (2C), 127.7, 127.6, 103.6 (2C), 103.4 (2C), 79.6, 74.94, 74.89, 66.1, 56.2 (2C), 56.0 (2C), 46.9, 34.9, 26.8 (3C), 19.1, 18.8, 8.2 ppm. Copies of ^1^H and ^13^C NMR could be found in Supplementary Materials.

### 3.5. Synthesis of Compound **11**

To a stirred solution of the tertiary alcohol **10** (840 mg, 1 mmol) in anhydrous CH_2_Cl_2_ (10 mL) at 0 °C under argon was added Et_3_SiH (349 mg, 3 mmol, 3 equiv). The resulting mixture was stirred for 5 min at this temperature, followed by the addition of BF_3_∙Et_2_O (0.5 mL, 3.6 mmol, 3.6 equiv) dropwise. After stirring for 5 min further, the reaction mixture was diluted with CH_2_Cl_2_ (80 mL), and the organic layers were washed with water (2 × 10 mL) and brine (10 mL), dried over Na_2_SO_4_, filtered and concentrated under reduced pressure. The resulting crude silyl ether (910 mg, 1 mmol) was dissolved in THF (10 mL) and cooled to 0 °C. TBAF (1.31 g, 5 mmol, 5 equiv) was then added and stirring was continued for 18 h at room temperature. The reaction mixture was then diluted with CH_2_Cl_2_ (80 mL) and poured into a separatory funnel. The organic layers were washed with water (2 × 10 mL) and brine (10 mL), dried over Na_2_SO_4_, filtered and concentrated under reduced pressure. The residue was purified by flash column chromatography (petroleum ether/EtOAc = 1:1) on silica gel to afford the primary alcohol **11** (527 mg, 90% yield) as a colorless oil. (*major isomer*) *R_f_* = 0.32 (petroleum ether/EtOAc = 1:1); ^1^H NMR (400 MHz, CDCl_3_): *δ* = 7.48–7.45 (m, 4H), 7.34–7.27 (m, 6H), 6.55 (s, 2H), 6.51 (s, 2H), 4.97 (s, 2H), 4.96 (s, 2H), 3.82 (s, 12H), 3.50–3.47 (m, 3H), 2.61–2.55 (m, 1H), 1.76–1.68 (m, 1H), 0.77 (d, *J* = 6.8 Hz, 3H), 0.68 (d, *J* = 6.8 Hz, 3H) ppm; ^13^C NMR (100 MHz, CDCl_3_): *δ* = 153.4 (2C), 153.3 (2C), 140.3, 139.9, 138.0, 136.1, 135.3, 132.9, 128.38 (2C), 128.35 (2C), 128.1 (4C), 127.69, 127.67, 105.1 (4C), 74.9 (2C), 66.8, 57.2, 56.2 (4C), 36.10, 36.08, 11.8, 9.8 ppm. Copies of ^1^H and ^13^C NMR could be found in Supplementary Materials.

### 3.6. Synthesis of Compound **12**

To a stirred solution of the primary alcohol **11** (176 mg, 0.3 mmol) in EtOAc (15 mL) at room temperature under argon was added IBX (1.00 g, 3.6 mmol, 12 equiv). The resulting mixture was stirred for 3.5 h at 80 °C, then cooled to room temperature, and filtered. The filter cake was washed with EtOAc (150 mL), and the combined filtrate was concentrated under reduced pressure. The residue was purified by flash column chromatography (petroleum ether/EtOAc = 2:1) on silica gel to afford the labile aldehyde **5** (154 mg, 88% yield) as a colorless oil.

To a stirred solution of the above aldehyde **5** (117 mg, 0.2 mmol) in toluene (5 mL) at room temperature under argon was added TsOH (19 mg, 0.1 mmol, 0.5 equiv). The reaction mixture was stirred for 1 h at this temperature, and quenched by saturated aqueous NaHCO_3_ (1 mL). The resulting mixture was diluted with CH_2_Cl_2_ (50 mL), and poured into a separatory funnel. The organic layers were washed with water (2 × 5 mL) and brine (5 mL), dried over Na_2_SO_4_, filtered and concentrated under reduced pressure. The residue was purified by flash column chromatography (petroleum ether/EtOAc = 2:1) on silica gel to afford benzyl-protected Sacidumlignan B (**12**, 104 mg, 92% yield) as a colorless oil. *R_f_* = 0.35 (petroleum ether/EtOAc = 4:1); ^1^H NMR (400 MHz, CDCl_3_): *δ* = 7.51 (d, *J* = 7.2 Hz, 2H), 7.47 (d, *J* = 7.2 Hz, 2H), 7.38–7.25 (m, 6H), 6.47 (s, 1H), 6.36 (s, 1H), 6.29 (s, 2H), 5.04 (s, 2H), 4.97 (s, 2H), 3.88 (s, 3H), 3.73 (s, 3H), 3.71 (s, 6H), 3.64 (d, *J* = 3.6 Hz, 1H), 2.44–2.38 (m, 1H), 1.84 (s, 3H), 1.08 (d, *J* = 7.2 Hz, 3H) ppm; ^13^C NMR (100 MHz, CDCl_3_): *δ* = 153.8 (2C), 152.0, 149.0, 140.8, 140.0, 138.7, 138.01, 137.95, 125.2, 131.1, 128.3 (2C), 128.22 (2C), 128.17 (2C), 128.0 (2C), 127.8, 127.7, 121.1, 115.0, 109.0, 104.8 (2C), 75.2, 74.9, 61.6, 56.0 (3C), 52.1, 41.6, 22.5, 18.6 ppm. Copies of ^1^H and ^13^C NMR could be found in Supplementary Materials.

### 3.7. Synthesis of Compound **13** and Sacidumlignan B (**2**)

Benzyl-protected Sacidumlignan B (**12**, 28 mg, 0.05 mmol) was dissolved in EtOH (2 mL) followed by the addition of 10% Pd/C (58 mg) at room temperature. The whole system with three-way Teflon stopcock connected to a standard balloon was evacuated and backfilled with H_2_, and this protocol was repeated three times. The resulting heterogeneous mixture was then allowed to stir under a positive pressure of hydrogen. After 2 h, the hydrogenation reaction was complete, and the reaction mixture was filtered through a pad of silica gel. The filter cake was washed with EtOAc (4 × 10 mL), and the combined filtrate was concentrated under reduced pressure. The residue was purified by flash column chromatography (petroleum ether/EtOAc = 1:1) on silica gel to afford the dihydroSacidumlignan B (**13**, 19 mg, 98% yield) as a white solid. *R_f_* = 0.31 (petroleum ether/EtOAc = 2:1); (*major isomer*) ^1^H NMR (400 MHz, CDCl_3_): *δ* = 6.32 (s, 2H), 5.99 (s, 1H), 5.44 (s, 1H), 5.40 (s, 1H), 3.89 (s, 3H), 3.85 (s, 6H), 3.60 (s, 3H), 3.36 (d, *J* = 10.4 Hz, 1H), 2.97 (dd, *J* = 16.8, 4.4 Hz, 1H), 2.35 (dd, *J* = 16.8, 11.6 Hz, 1H), 1.60–1.44 (m, 2H), 1.11 (d, *J* = 6.4 Hz, 3H), 0.85 (d, *J* = 6.4 Hz, 3H) ppm; ^13^C NMR (100 MHz, CDCl_3_): *δ* = 146.8 (2C), 145.4, 143.5, 137.5, 136.3, 132.8, 131.7, 123.4, 107.8, 106.0 (2C), 60.1, 56.3 (2C), 56.1, 55.1, 43.4, 35.1, 32.6, 20.1, 17.2 ppm. Copies of ^1^H and ^13^C NMR could be found in Supplementary Materials.

The above benzyl-protected Sacidumlignan B (**12**, 57 mg, 0.1 mmol) was dissolved in anhydrous CH_2_Cl_2_ (2 mL), and cooled to −45 °C followed by the addition of a solution of TiCl_4_ (0.5 mmol, 5 equiv) in CH_2_Cl_2_ (2.0 M, 0.25 mL). The reaction mixture was stirred for 2 h at −45 °C, and quenched by saturated aqueous NaHCO_3_ (0.5 mL). The resulting mixture was then diluted with CH_2_Cl_2_ (50 mL) and poured into a separatory funnel. The organic layers were washed with water (2 × 5 mL) and brine (5 mL), dried over Na_2_SO_4_, filtered and concentrated under reduced pressure. The residue was purified by flash column chromatography (petroleum ether/EtOAc = 1:1) on silica gel to afford the racemic Sacidumlignan B (**2**, 18 mg, 47% yield) as a white solid. m.p. = 120.4 °C. *R_f_* = 0.30 (petroleum ether/EtOAc = 2:1); ^1^H NMR (400 MHz, CDCl_3_): *δ* = 6.45 (s, 1H), 6.35 (s, 1H), 6.30 (s, 2H), 5.43 (s, 1H), 5.34 (s, 1H), 3.88 (s, 3H), 3.79 (s, 3H), 3.78 (s, 6H), 3.61 (d, *J* = 3.2 Hz, 1H), 2.37 (dq, *J* = 6.8, 3.6 Hz, 1H), 1.82 (s, 3H), 1.07 (d, *J* = 6.8 Hz, 3H) ppm; ^13^C NMR (100 MHz, CDCl_3_): *δ* = 146.7 (2C), 145.9, 142.4, 139.1, 137.0, 136.6, 132.9, 126.7, 120.8, 114.9, 108.1, 104.3 (2C), 61.3, 56.2 (2C), 51.7 (2C), 41.9, 22.6, 18.7 ppm. Copies of ^1^H and ^13^C NMR could be found in Supplementary Materials.

### 3.8. Alternative Synthesis of Sacidumlignan B (**2**)

The above aldehyde **5** (58 mg, 0.1 mmol) was dissolved in EtOH (2 mL) followed by the addition of 10% Pd/C (116 mg) at room temperature. The whole system was evacuated and backfilled with H_2_, and this protocol was repeated three times. The resulting heterogeneous mixture was then allowed to stir under a positive pressure of hydrogen. After 2 h, the hydrogenation reaction finished, and the reaction mixture was filtered through a short plug of silica gel. The filter cake was washed with EtOAc (4 × 10 mL), and the combined filtrate was concentrated under reduced pressure. The residue was purified by flash column chromatography (petroleum ether/EtOAc = 1:2) on silica gel to afford the labile phenol-aldehyde **14** (40 mg) as a colorless oil.

To a stirred solution of the above phenol-aldehyde **14** (40 mg, 0.1 mmol) in toluene (2 mL) at room temperature under argon was added TsOH (10 mg, 0.05 mmol, 0.5 equiv). The reaction mixture was stirred for 1 h at this temperature, and quenched by saturated aqueous NaHCO_3_ (1 mL). The resulting mixture was diluted with CH_2_Cl_2_ (50 mL), and poured into a separatory funnel. The organic layers were washed with water (2 × 5 mL) and brine (5 mL), dried over Na_2_SO_4_, filtered and concentrated under reduced pressure. The residue was purified by flash column chromatography (petroleum ether/EtOAc = 1:1) on silica gel to afford the racemic Sacidumlignan B (**2**, 33 mg, 85% yield) as a white solid This sample demonstrated identical NMR data to those from previous synthesis.

## 4. Conclusions

An efficient total synthesis of 2,7′-cyclolignan sacidumlignan B was realized by the strategic utilization of a formal reductive coupling between the easily-available diaryl ketone and the crotyl bromide. The facile construction of dihydronaphthalene skeleton allows the further synthetic investigation towards other more challenging cyclolignans [15,16,17,18], and the related progress will be reported in due course.

## Figures and Tables

**Table 1:** Removal of Benzyl Groups.

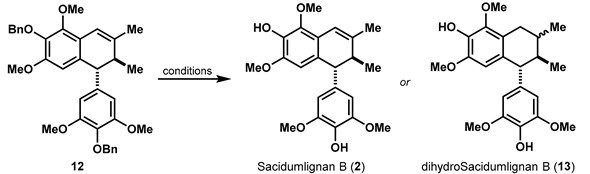					
Entry	Reagents	Solvent	T/°C	2 Yield (%)	13 Yield (%)
1	Pd-C/H2	EtOH	20	0	98
2	AlCl3	Me2S	22	0	0
3	FeCl3	DCM	22	0	0
4	TiCl4	DCM	0	15	0
5	TiCl4	DCM	−45	47	0
6	TiCl4	DCM	−78	39	0

## Data Availability

Not applicable.

## Supplementary Materials

Copies of ^1^H and ^13^C NMR are available online at https://www.mdpi.com/article/10.3390/molecules27185775/s1.

## Sample Availability

Samples of the compounds are not available from the authors.

## References

[1] Gan L.-S., Yang S.-P., Fan C.-Q., Yue J.-M. (2005). Lignans and Their Degraded Derivatives from *Sarcostemma acidum*. J. Nat. Prod..

[2] Zhang H.-Q., Yan C.-X., Xiao J., Wang Y.-W., Peng Y. (2022). Recent Advances in the Total Synthesis of 2,7′-cyclolignans. Org. Biomol. Chem..

[3] Fang X., Hu X. (2018). Advances in the Synthesis of Lignan Natural Products. Molecules.

[4] Rout J.K., Ramana C.V. (2012). Total Synthesis of (−)-Sacidumlignans B and D. J. Org. Chem..

[5] Zhang J.-J., Yan C.-S., Peng Y., Luo Z.-B., Xu X.-B., Wang Y.-W. (2013). Total Synthesis of (±)-Sacidumlignans D and A through Ueno−Stork Radical Cyclization Reaction. Org. Biomol. Chem..

[6] Luo Z.-B., Wang Y.-W., Peng Y. (2020). Base-Promoted Addition of DMA with 1,1-Diarylethylenes: Application to a Total Synthesis of (−)-Sacidumlignan B. Org. Biomol. Chem..

[7] Xie C., Bai D., Huang S.-H., Jia X., Hong R. (2014). Kinetic Resolution of Diols via Etherification Catalyzed by a Chiral Phosphoric Acid: Concise Synthesis of (+)-Sacidumlignan D. Asian J. Org. Chem..

[8] Wang Y., Zhu L., Zhang Y., Hong R. (2011). Bioinspired and Concise Synthesis of (±)-Stemoamide. Angew. Chem. Int. Ed..

[9] Kim H., Wooten C.M., Park Y., Hong J. (2007). Stereoselective Synthesis of Tetrahydrofuran Lignans via BF_3_·OEt_2_-Promoted Reductive Deoxygenation/Epimerization of Cyclic Hemiketal:  Synthesis of (−)-Odoratisol C, (−)-Futokadsurin A, (−)-Veraguensin, (+)-Fragransin A2, (+)-Galbelgin, and (+)-Talaumidin. Org. Lett..

[10] Shang Y., Xiao J., Wang Y.-W., Peng Y. (2021). Advances on Asymmetric Construction of Diarylmethine Stereocenters. Acta Chim. Sinica.

[11] Akiyama T., Hirofuji H., Ozaki S. (1991). AlCl_3_-N,N-Dimethylaniline: A New Benzyl and Allyl Ether Cleavage Reagent. Tetrahedron Lett..

[12] Jempty T., Gogins K.A.Z., Mazur Y., Miller L. (1981). FeCl_3_/SiO_2_ Reacts as Oxidant or Lewis Acid with Phenol Ethers. J. Org. Chem..

[13] Hori H., Nishida Y., Ohrui H., Meguro H. (1989). Regioselective De-*O*-benzylation with Lewis Acid. J. Org. Chem..

[14] Loh T.-P., Cao G.-Q., Pei J. (1998). Studies Towards Total Synthesis of Antillatoxin: Synthesis of C1-C11 Fragment. Tetrahedron Lett..

[15] Xiao J., Cong X.-W., Yang G.-Z., Wang Y.-W., Peng Y. (2018). Divergent Asymmetric Syntheses of Podophyllotoxin and Related Family Members via Stereoselective Reductive Ni-catalysis. Org. Lett..

[16] Xiao J., Nan G., Wang Y.-W., Peng Y. (2018). Concise Syntheses of (+)-β- and γ-Apopicropodophyllins, and Dehydrodesoxypodophyllotoxin. Molecules.

[17] Fang X., Shen L., Hu X. (2018). Asymmetric Total Synthesis of (+)-Ovafolinins A and B. Chem. Commun..

[18] Davidson S.J., Barker D. (2017). Total Synthesis of Ovafolinins A and B: Unique Polycyclic Benzoxepin Lignans through a Cascade Cyclization. Angew. Chem. Int. Ed..

