# Supplementary material for: A Concise Synthesis of Sacidumlignan B
Source: Molecules. 2022 Sep 7;27(18):5775. doi: 10.3390/molecules27185775 (PMC9500800; doi:10.3390/molecules27185775)
Supplement: Supplementary file 1 [file molecules-27-05775-s001.zip › molecules-1890807-supplementary.pdf]

molecules

Supporting Information for

A Concise Synthesis of Sacidumlignan B

Zhiyuan Zhuang [1], Zhenbiao Luo [1], Sichen Yao [2], Yawen Wang [2,*] and Yu Peng [2,*]

[1] State Key Laboratory of Applied Organic Chemistry, College of Chemistry and Chemical Engineering, Lanzhou University, Lanzhou 73000, China; zhuangzhy17@lzu.edu.cn (Z.Z.); luozhb12@lzu.edu.cn (Z.L.)
[2] Sichuan Engineering Research Center for Biomimetic Synthesis of Natural Drugs, School of Life Science and Engineering, Southwest Jiaotong University, Chengdu 610031, China; yaosichen@my.swjtu.edu.cn
* Correspondence: ywwang@swjtu.edu.cn (Y.W.); pengyu@swjtu.edu.cn (Y.P.)

Table of Contents

- ^{1}H NMR and ^{13}C NMR spectra of **9**.....S2-S3
- ^{1}H NMR and ^{13}C NMR spectra of **10** (*major isomer*).....S4-S5
- ^{1}H NMR and ^{13}C NMR spectra of **11** (*major isomer*).....S6-S7
- ^{1}H NMR and ^{13}C NMR spectra of **12**.....S8-S9
- ^{1}H NMR and ^{13}C NMR spectra of **13**.....S10-S11
- ^{1}H NMR and ^{13}C NMR spectra of Sacidumlignan B (**2**).....S12-S13

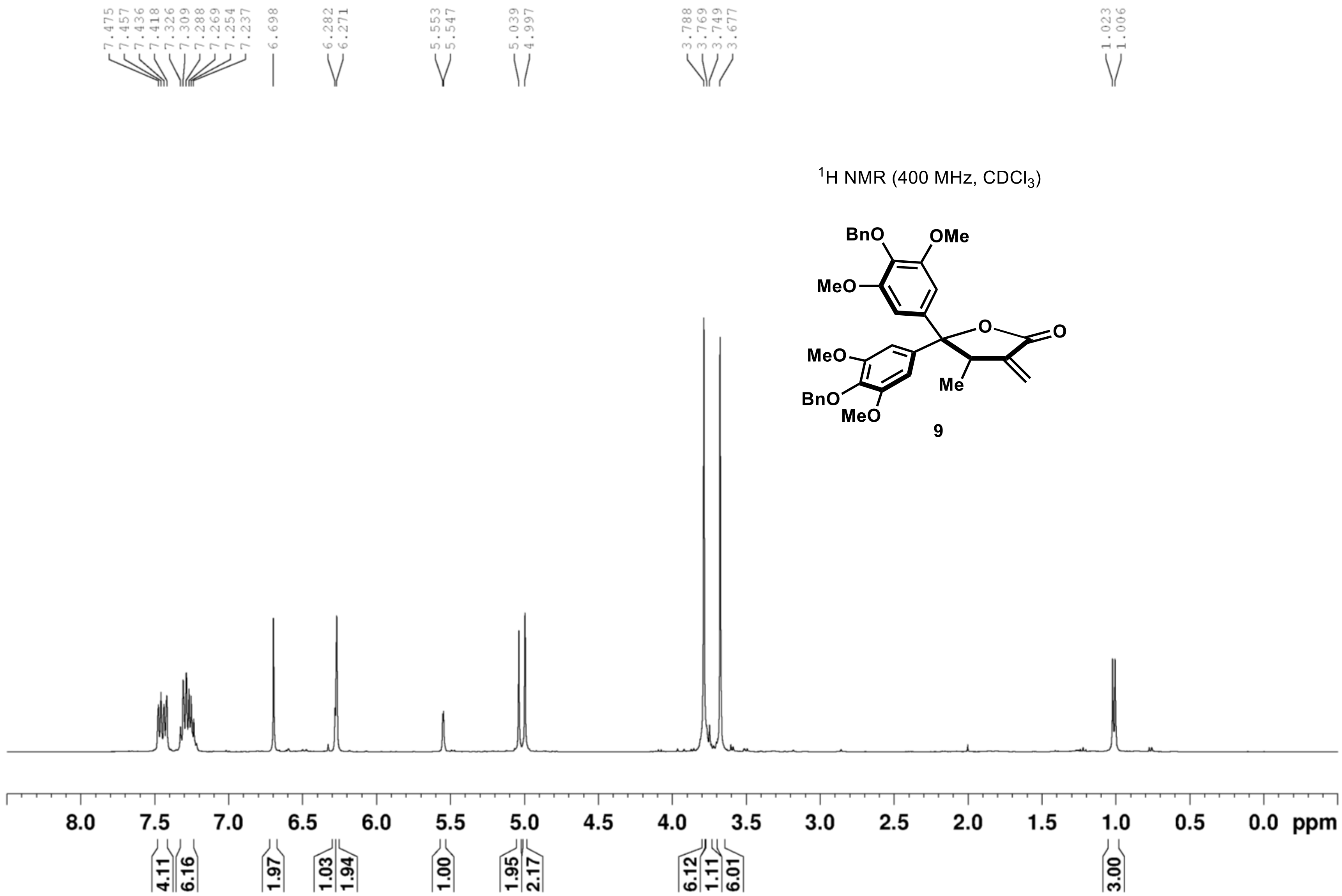

^1H NMR (400 MHz, $CDCl_3$)

9

^{13}C NMR (100 MHz, $CDCl_3$)

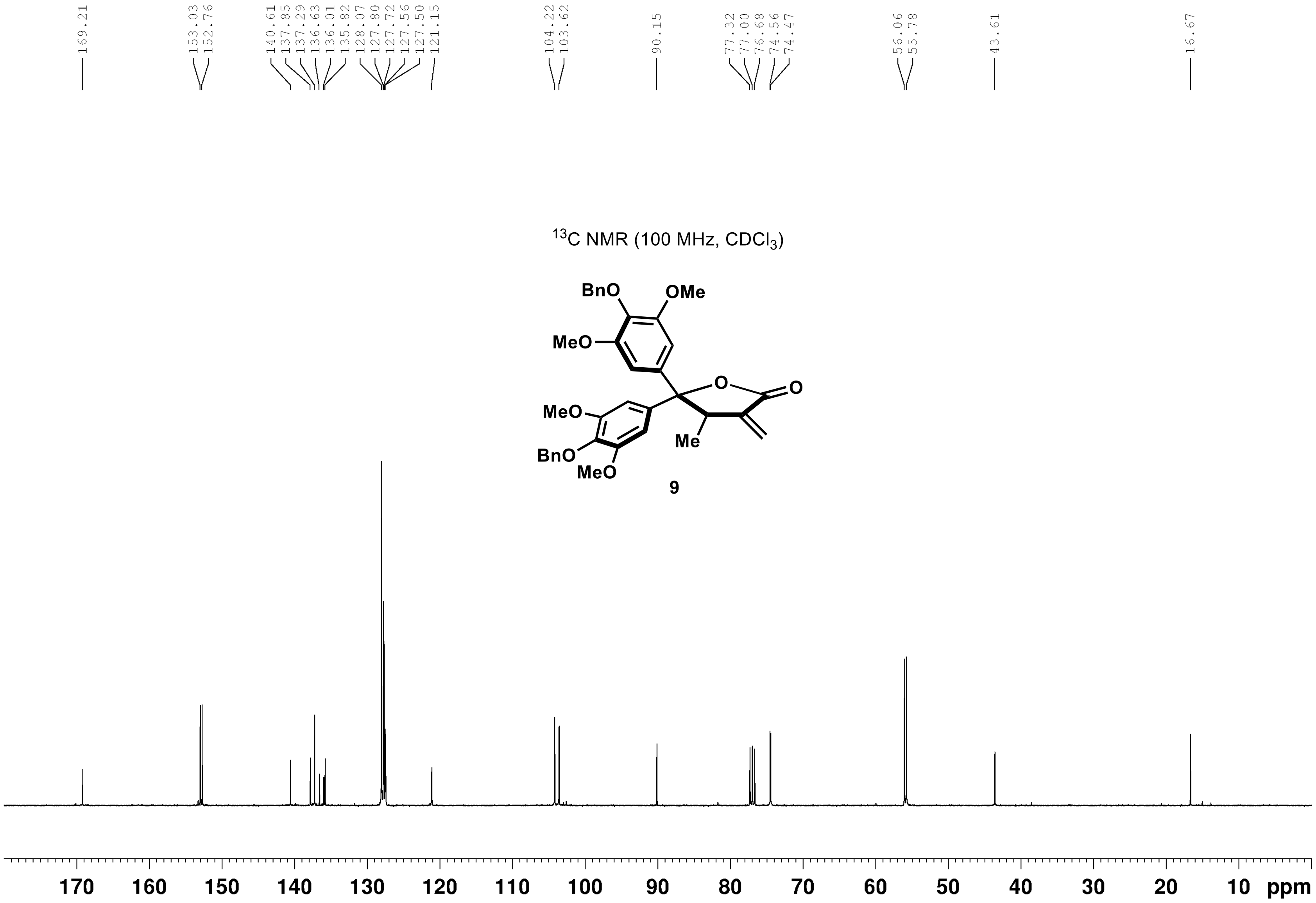

9

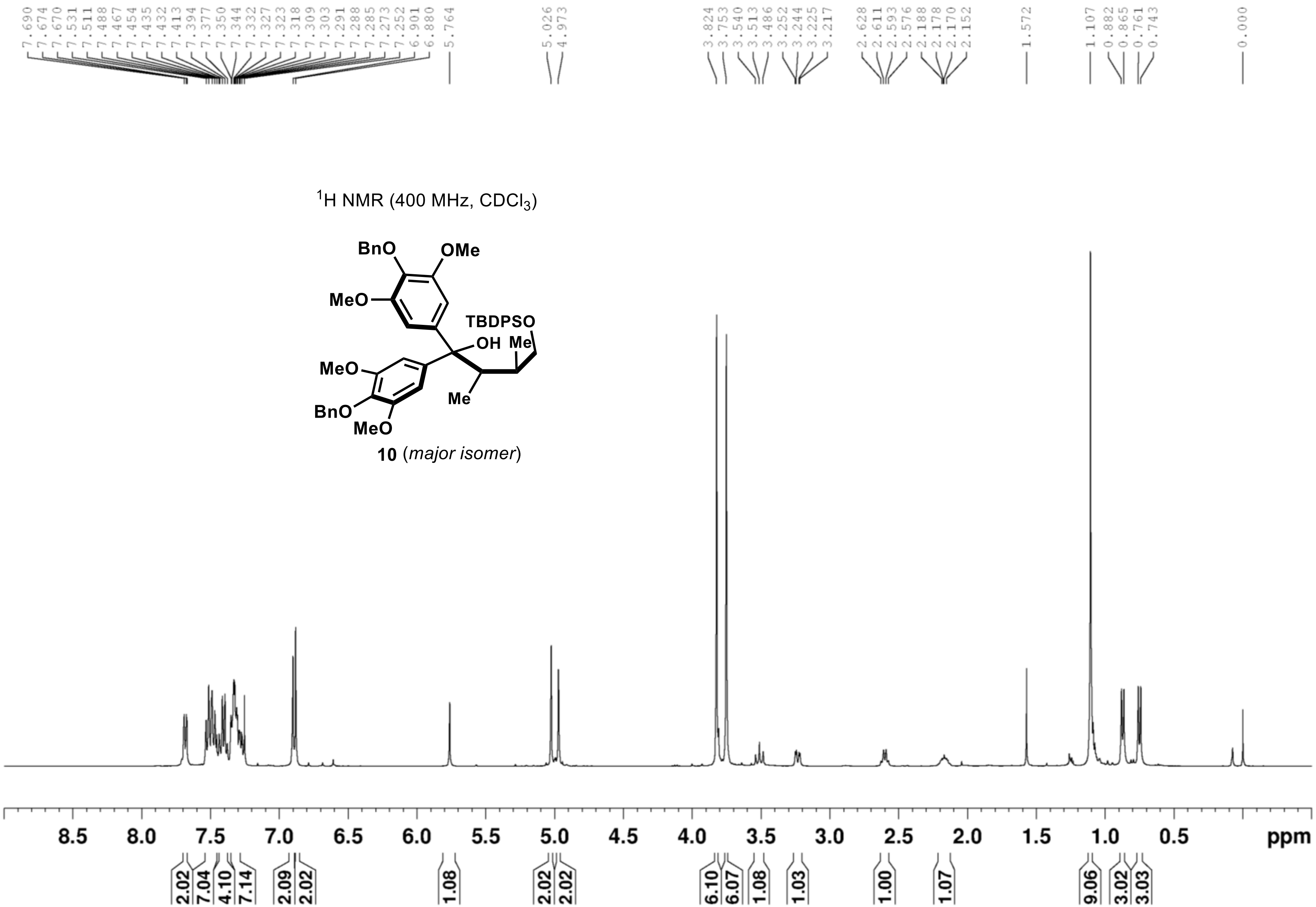

^1H NMR (400 MHz, $CDCl_3$)

10 (*major isomer*)

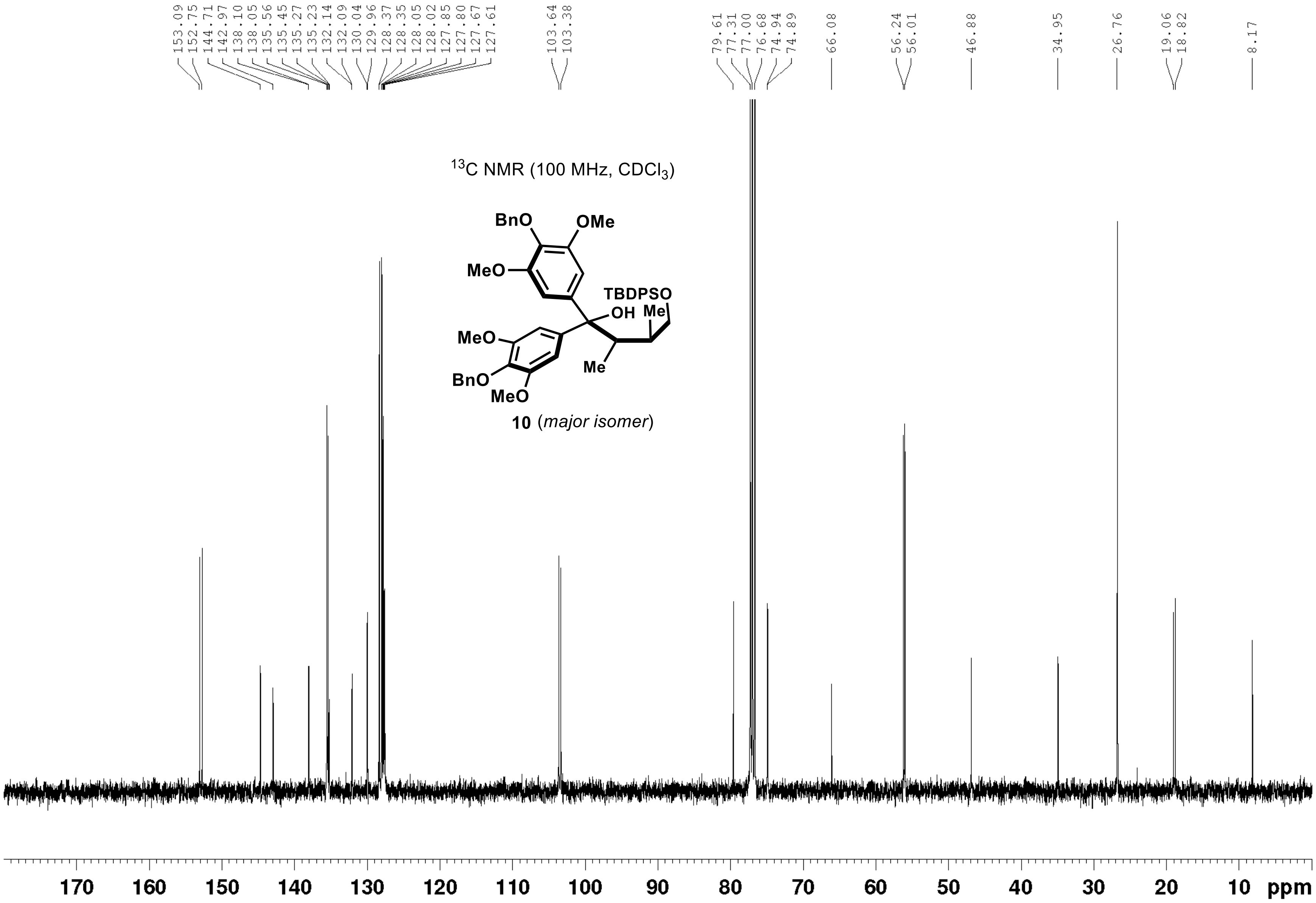
153.09
152.75
144.71
142.97
138.10
138.05
135.56
135.45
135.27
135.23
132.14
132.09
130.04
129.96
128.37
128.35
128.05
128.02
127.85
127.80
127.67
127.61
103.64
103.38
79.61
77.31
77.00
76.68
74.94
74.89
66.08
56.24
56.01
46.88
34.95
26.76
19.06
18.82
8.17
13C NMR (100 MHz, CDCl3)
BnO
OMe
MeO
TBDPSO
OH
Me
MeO
BnO
MeO
Me
10 (major isomer)
170
160
150
140
130
120
110
100
90
80
70
60
50
40
30
20
10
ppm

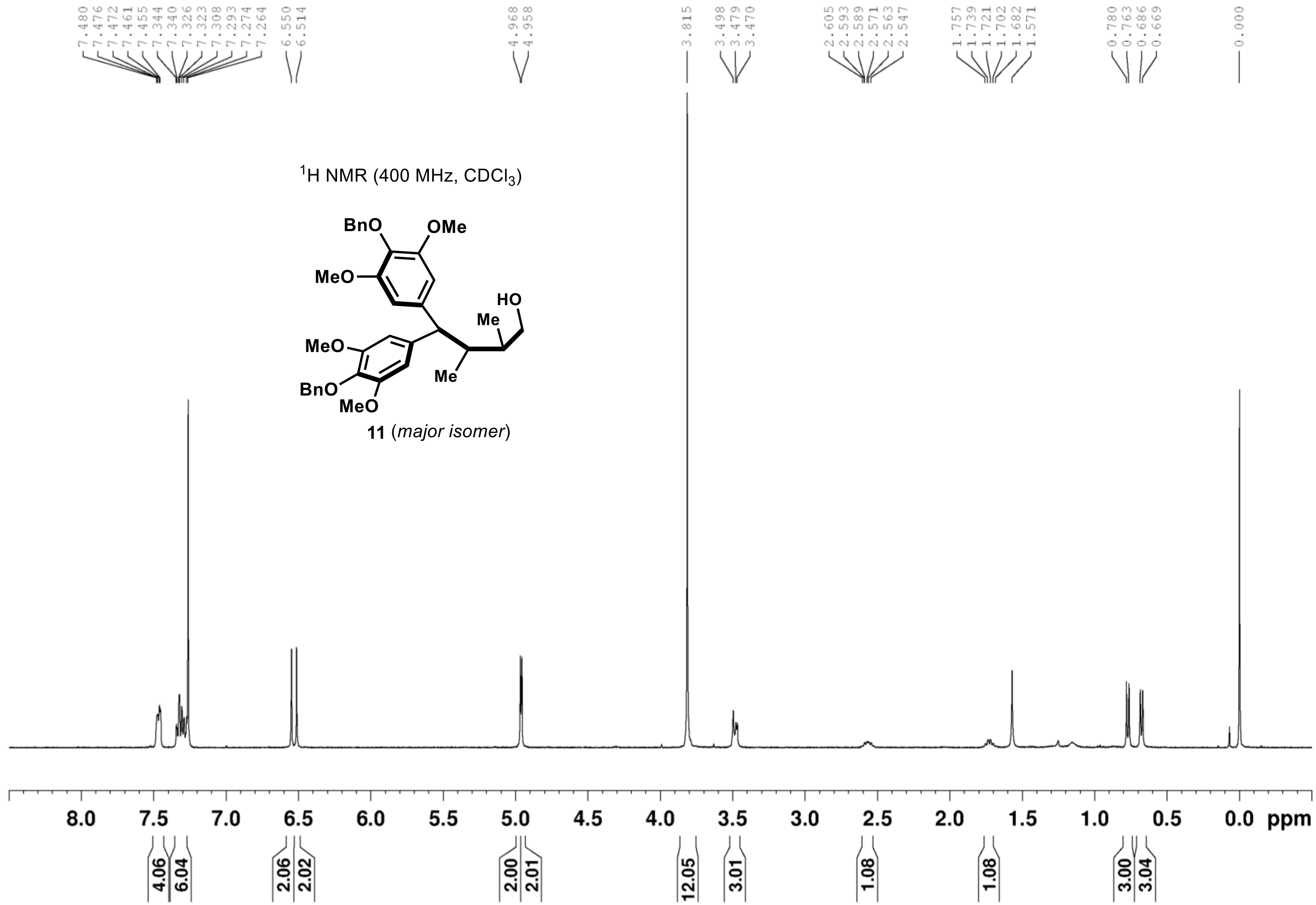
7.480
7.476
7.472
7.461
7.455
7.344
7.340
7.326
7.323
7.308
7.293
7.274
7.264
6.550
6.514
4.968
4.958
3.815
3.498
3.479
3.470
2.605
2.593
2.589
2.571
2.563
2.547
1.757
1.739
1.721
1.702
1.682
1.571
0.780
0.763
0.686
0.669
0.000
^{1}H NMR (400 MHz, $CDCl_3$)
BnO
OMe
MeO
HO
Me
MeO
Me
BnO
MeO
11 (*major isomer*)
8.0
7.5
7.0
6.5
6.0
5.5
5.0
4.5
4.0
3.5
3.0
2.5
2.0
1.5
1.0
0.5
0.0
ppm
4.06
6.04
2.06
2.02
2.00
2.01
12.05
3.01
1.08
1.08
3.00
3.04

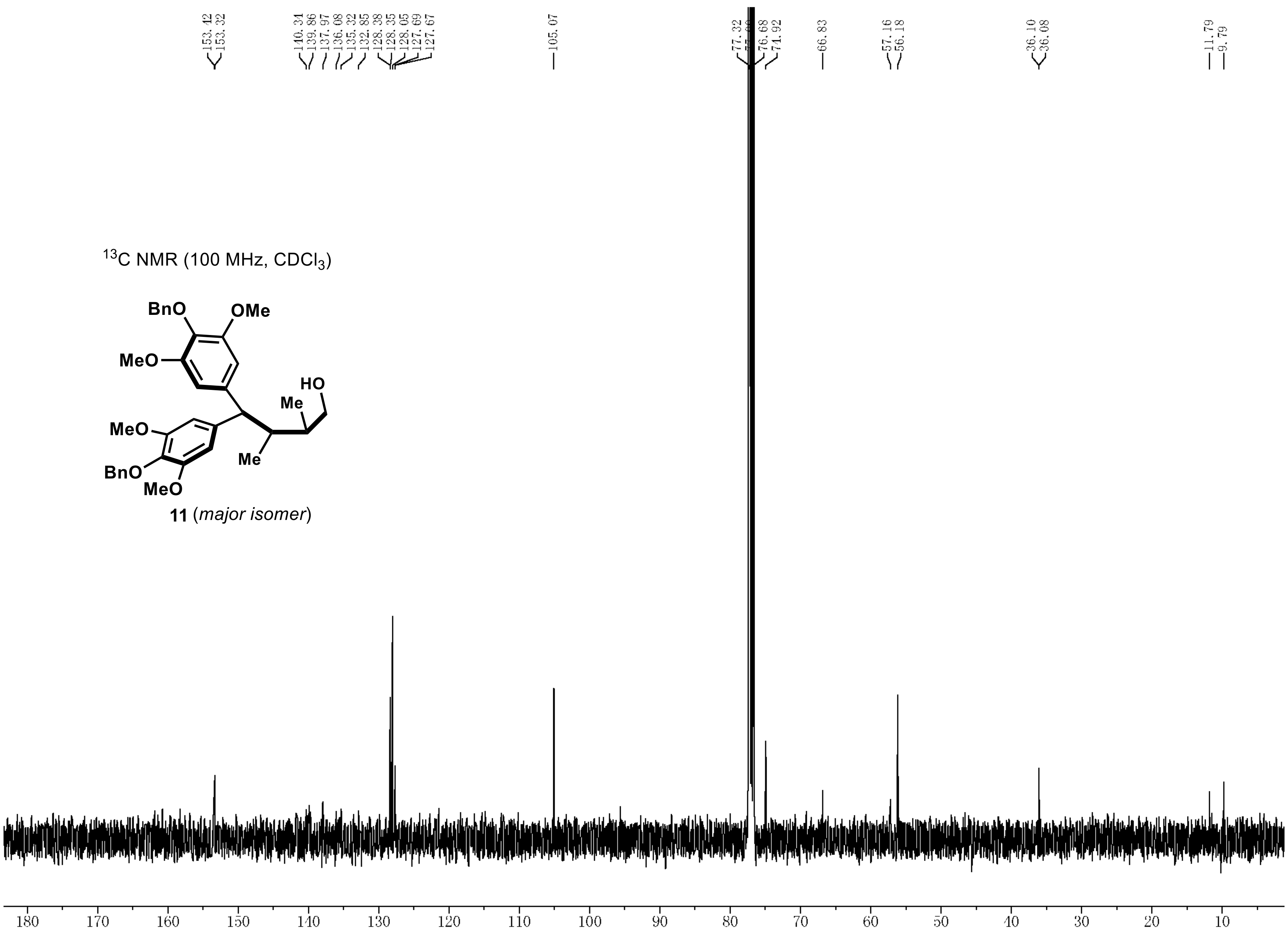
153.42
153.32
140.31
139.86
137.97
136.08
135.32
132.85
128.38
128.35
128.05
127.69
127.67
105.07
77.32
76.68
74.92
66.83
57.16
56.18
36.10
36.08
11.79
9.79
^{13}C NMR (100 MHz, $CDCl_3$)
BnO
OMe
MeO
HO
Me
MeO
Me
BnO
MeO
11 (*major isomer*)
180
170
160
150
140
130
120
110
100
90
80
70
60
50
40
30
20
10

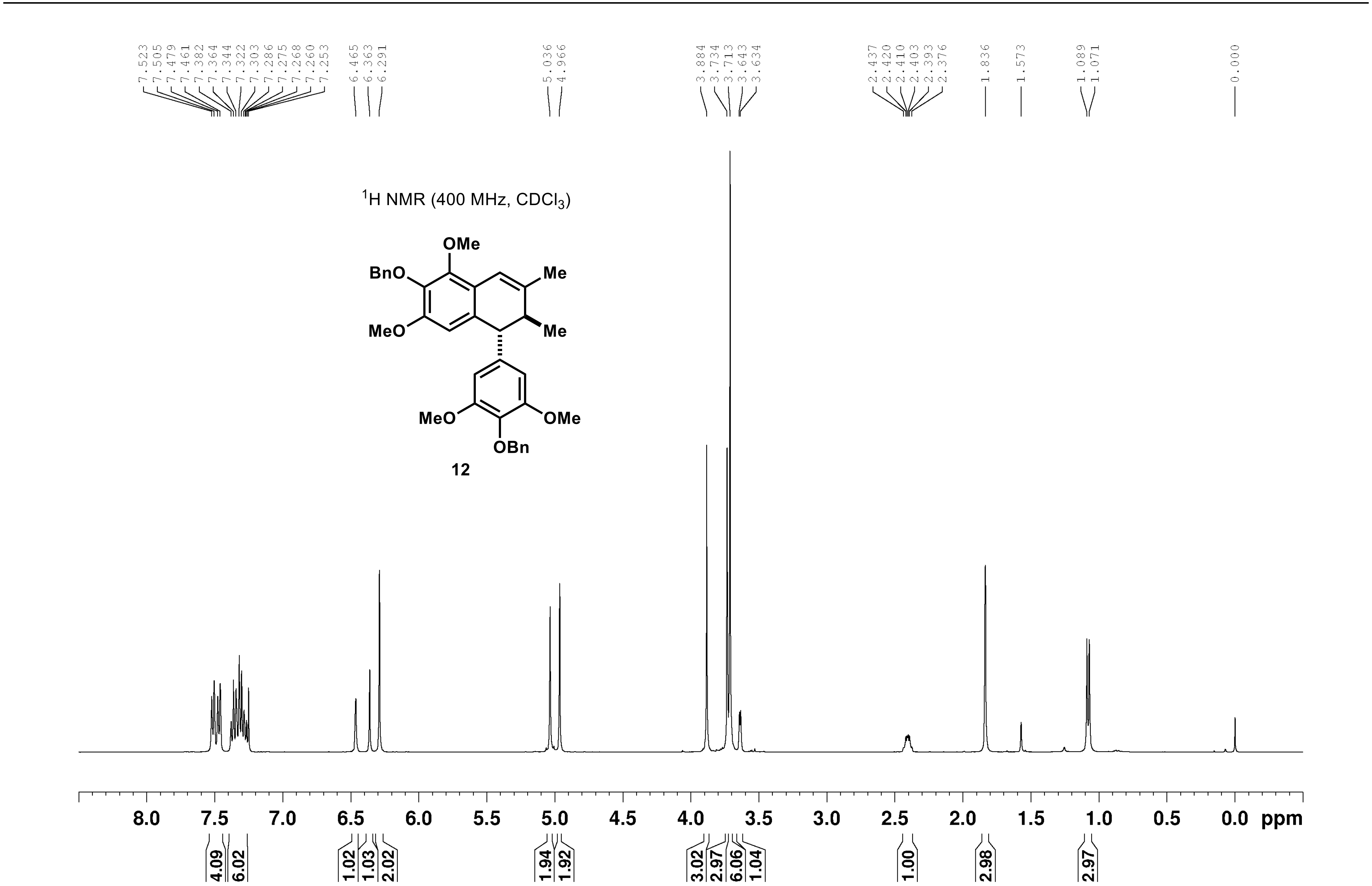

1H NMR (400 MHz, CDCl3)
OMe
BnO
Me
MeO
Me
MeO
OMe
OBn
12
7.523 7.505 7.479 7.461 7.382 7.364 7.344 7.322 7.303 7.286 7.275 7.268 7.260 7.253
6.465 6.363 6.291
5.036 4.966
3.884 3.734 3.713 3.643 3.634
2.437 2.420 2.410 2.403 2.393 2.376
1.836
1.573
1.089 1.071
0.000
8.0 7.5 7.0 6.5 6.0 5.5 5.0 4.5 4.0 3.5 3.0 2.5 2.0 1.5 1.0 0.5 0.0 ppm
4.09 6.02 1.02 1.03 2.02 1.94 1.92 3.02 2.97 6.06 1.04 1.00 2.98 2.97

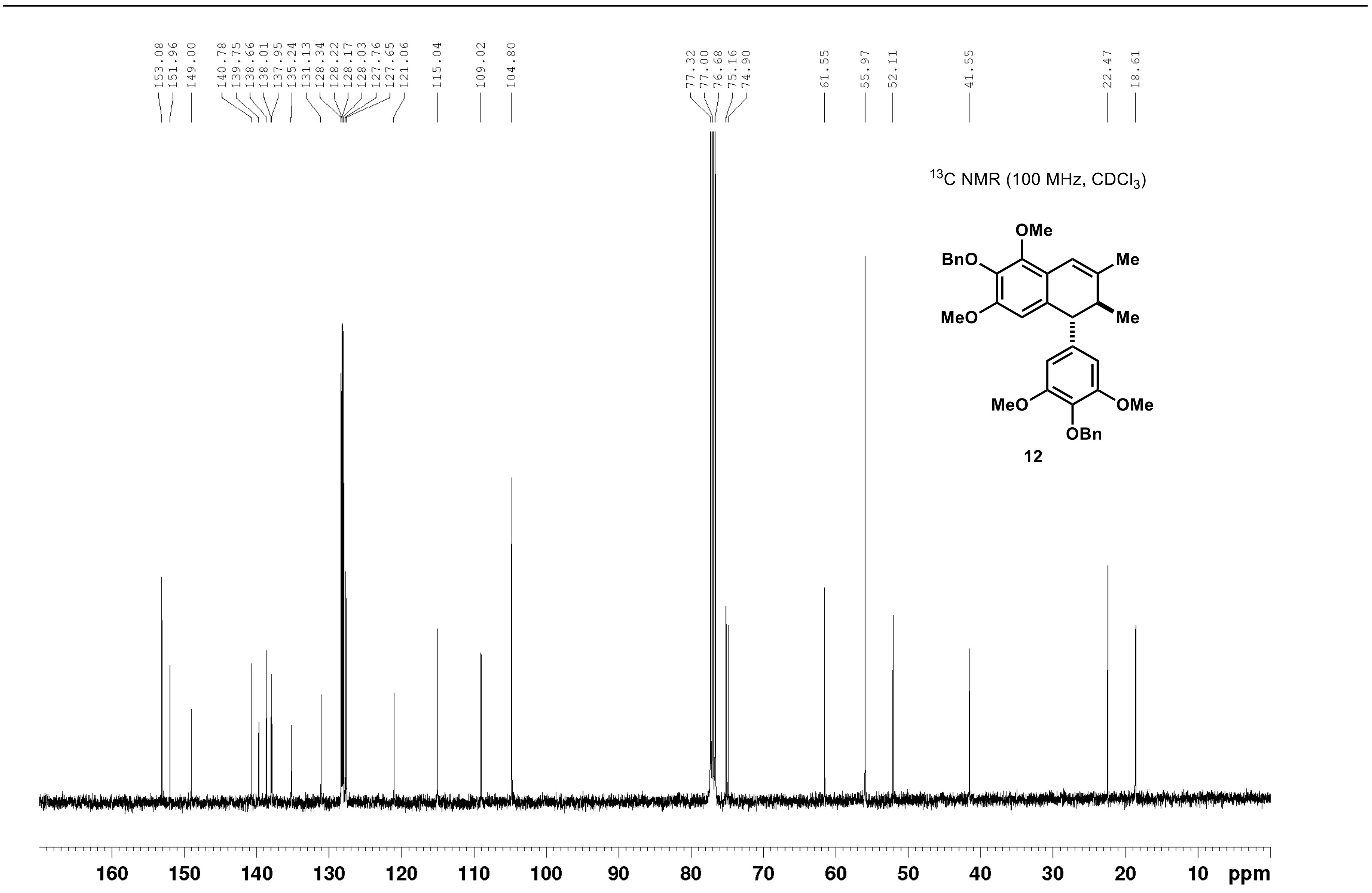
153.08
151.96
149.00
140.78
139.75
138.66
138.01
137.95
135.24
131.13
128.34
128.22
128.17
128.03
127.76
127.65
121.06
115.04
109.02
104.80
77.32
77.00
76.68
75.16
74.90
61.55
55.97
52.11
41.55
22.47
18.61
^{13}C NMR (100 MHz, $CDCl_3$)
OMe
BnO
Me
MeO
Me
MeO
OMe
OBn
12
160
150
140
130
120
110
100
90
80
70
60
50
40
30
20
10
ppm

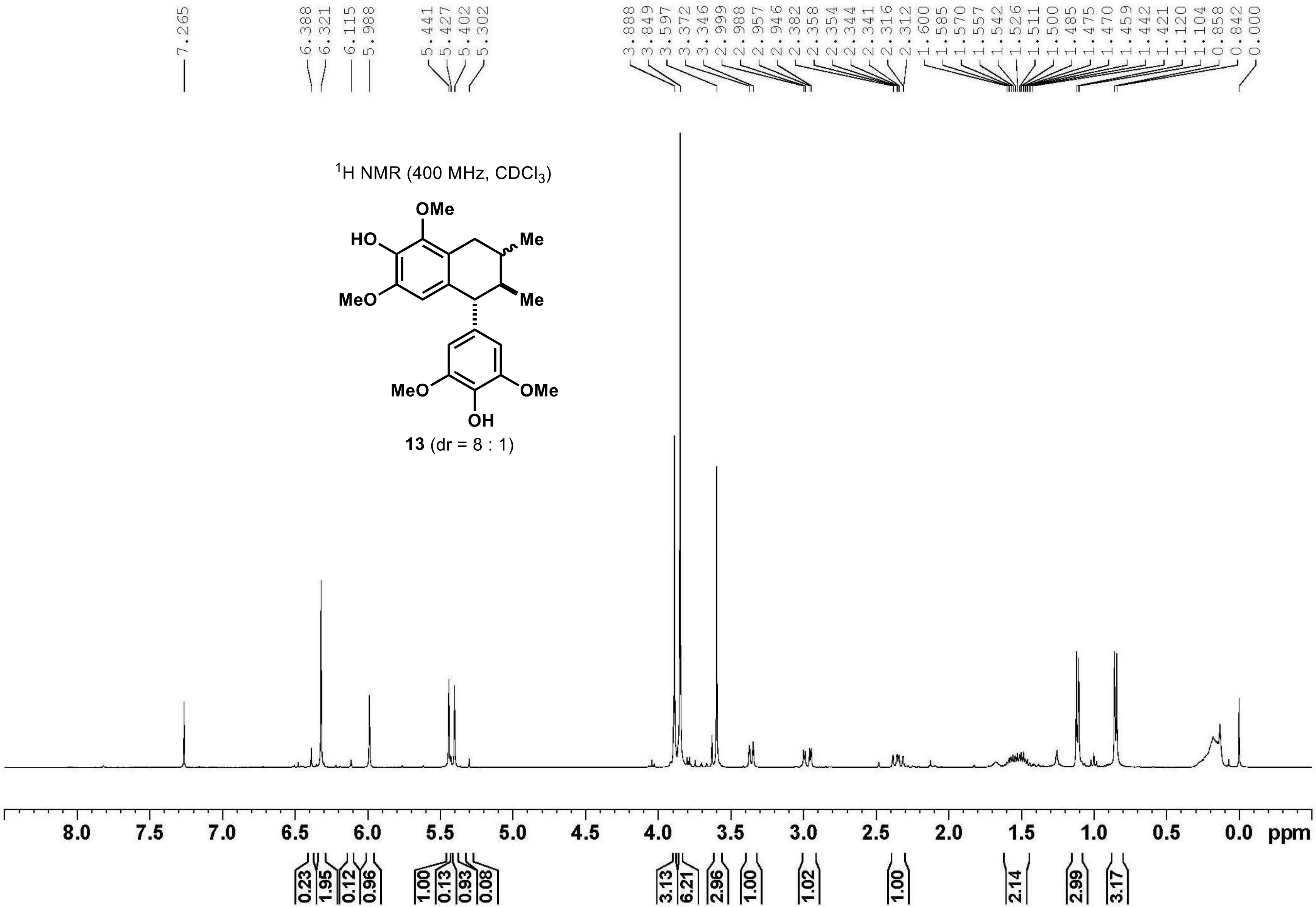

7.265
6.388
6.321
6.115
5.988
5.441
5.427
5.402
5.302
3.888
3.849
3.597
3.372
3.346
2.999
2.988
2.957
2.946
2.382
2.358
2.354
2.344
2.341
2.316
2.312
1.600
1.585
1.570
1.557
1.542
1.526
1.511
1.500
1.485
1.475
1.470
1.459
1.442
1.421
1.120
1.104
0.858
0.842
0.000
¹H NMR (400 MHz, CDCl₃)
OMe
HO
Me
MeO
Me
MeO
OMe
OH
13 (dr = 8 : 1)
8.0
7.5
7.0
6.5
6.0
5.5
5.0
4.5
4.0
3.5
3.0
2.5
2.0
1.5
1.0
0.5
0.0
ppm
0.23
1.95
0.12
0.96
1.00
0.13
0.93
0.08
3.13
6.21
2.96
1.00
1.02
1.00
2.14
2.99
3.17

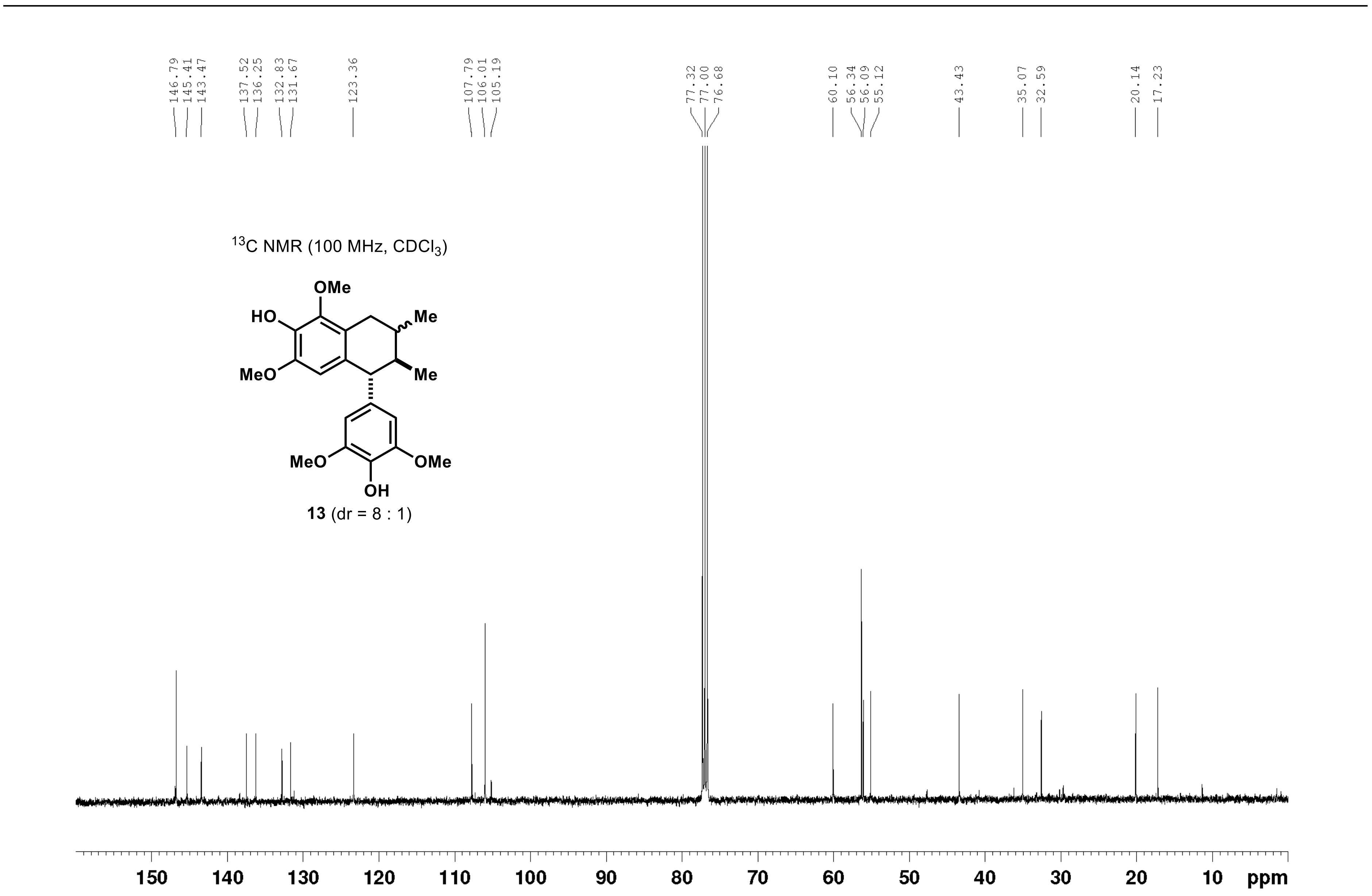

146.79
145.41
143.47
137.52
136.25
132.83
131.67
123.36
107.79
106.01
105.19
77.32
77.00
76.68
60.10
56.34
56.09
55.12
43.43
35.07
32.59
20.14
17.23
^{13}C NMR (100 MHz, $CDCl_3$)
OMe
HO
Me
MeO
Me
MeO
OMe
OH
13 (dr = 8 : 1)
150
140
130
120
110
100
90
80
70
60
50
40
30
20
10
ppm

^1H NMR (400 MHz, $CDCl_3$)

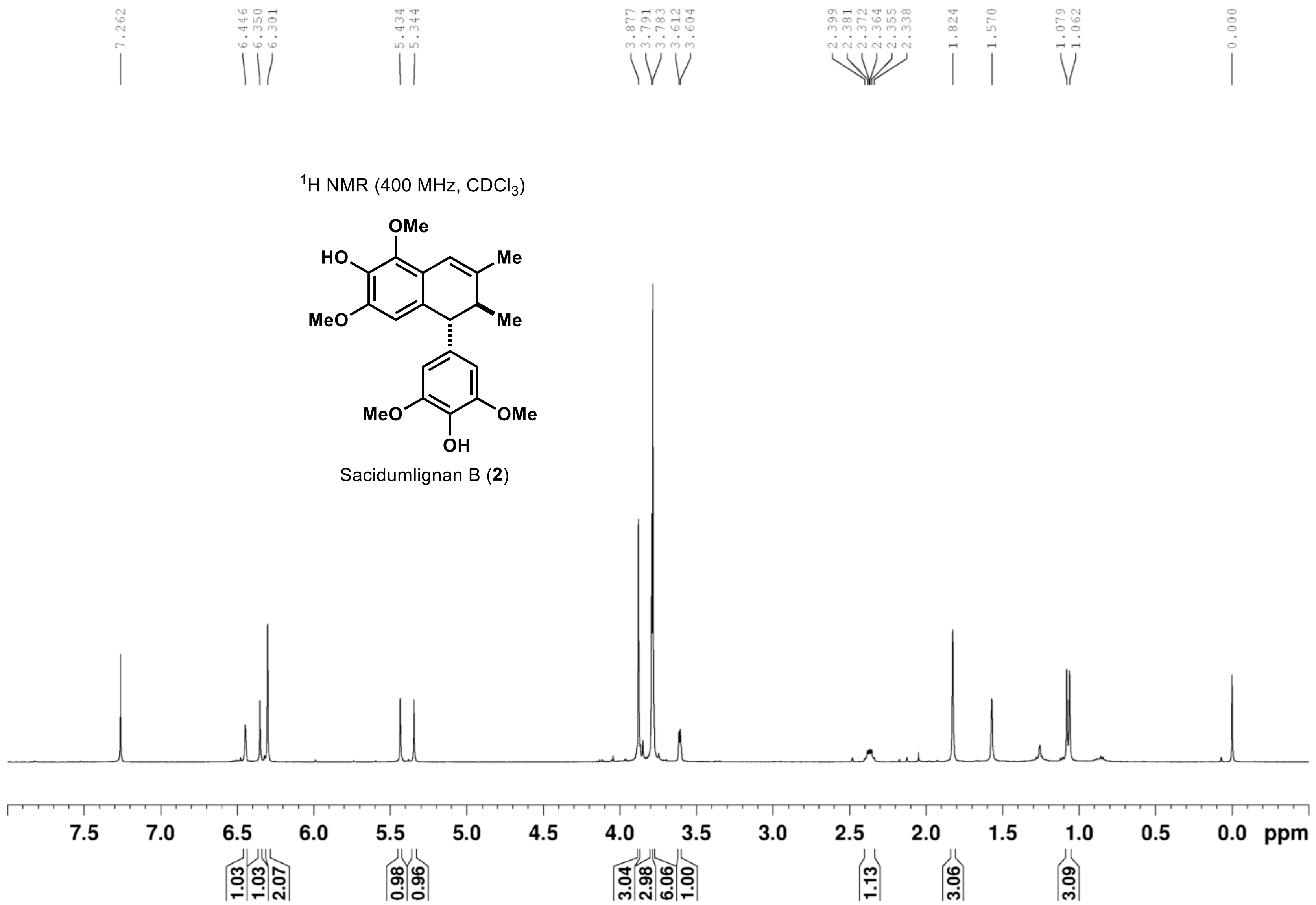

Sacidumlignan B (**2**)

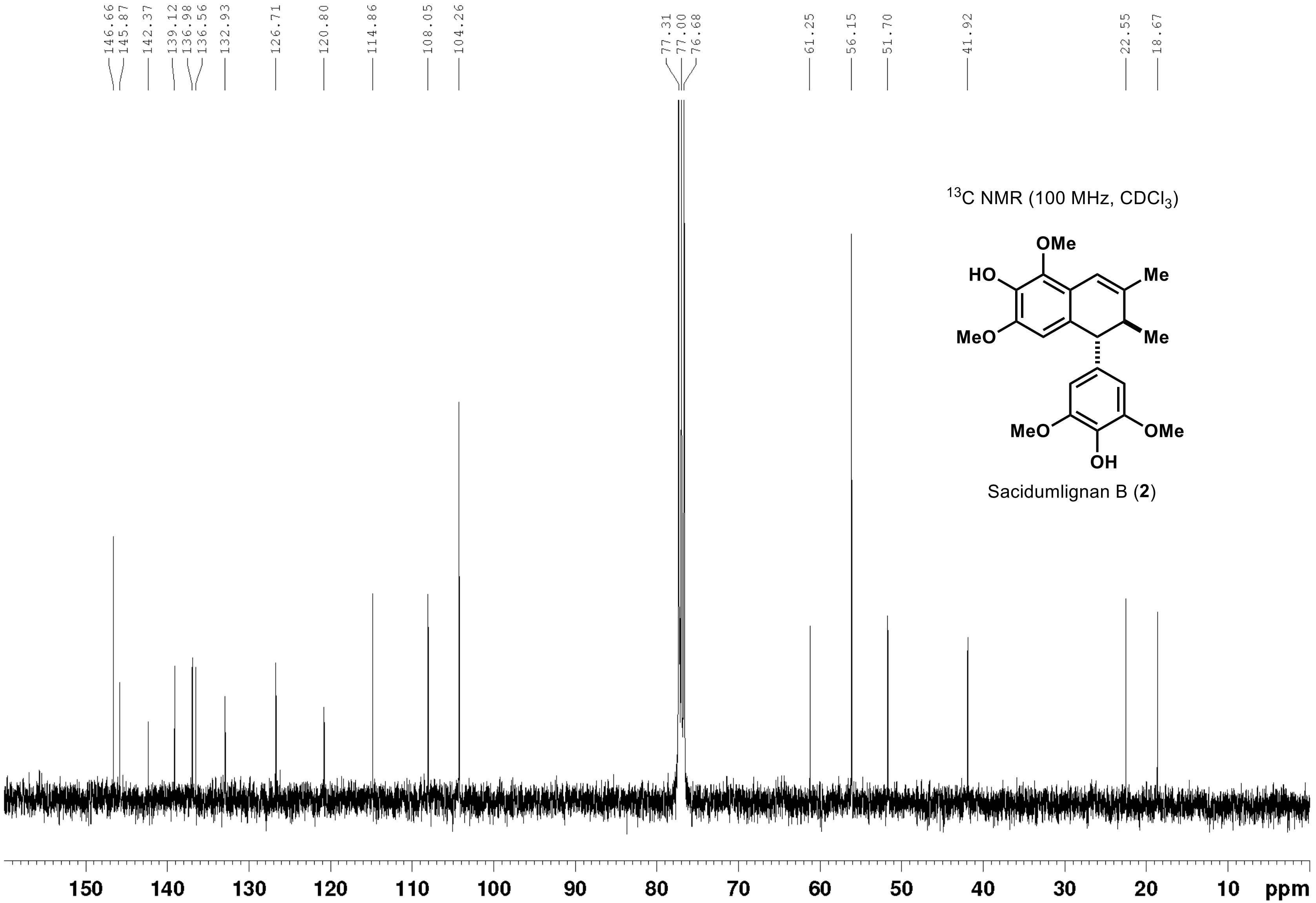

Sacidumlignan B (**2**)
